# 4013 Does dietary fat composition predict short-term elevations in lipid levels in adults on a modified Atkins diet?

**DOI:** 10.1017/cts.2020.122

**Published:** 2020-07-29

**Authors:** Tanya McDonald, Bobbie J Henry-Barron, Diane Vizthum, Mackenzie C. Cervenka

**Affiliations:** 1 Johns Hopkins University School of Medicine; 2 Institute for Clinical and Translational Research, JHU

## Abstract

OBJECTIVES/GOALS: The modified Atkins diet (MAD) is used in the management of drug-resistant epilepsy in adults. Some patients on MAD show an increase in serum levels of total cholesterol and low-density lipoprotein (LDL) cholesterol. We explored whether dietary fat composition predicts short-term elevations in serum lipid levels in diet-naïve adults who begin MAD. METHODS/STUDY POPULATION: Participants self-reported their diet intake with 3-day food records at baseline, 1 month and 2 months. Food records were analyzed using Nutrition Data System for Research software. Fasting serum levels of total cholesterol (TC), high-density lipoprotein (HDL) cholesterol, and triglycerides were also collected and LDL level calculated at baseline, 1 month, and 2 months. RESULTS/ANTICIPATED RESULTS: 38 patients submitted complete food records at each study visit (baseline, 1 month, and 2 month). Compared to baseline diet intake, there was a significant reduction in daily carbohydrate intake at 1 and 2 months months (p<0.001). There was also a significant increase in daily saturated fatty acid (SFA) intake at 1 and 2 months (p<0.001), daily mono-unsaturated fatty acid (MUFA) intake at 1 and 2 months (p<0.001), and daily cholesterol intake at 1 month (p<0.05) and 2 months (p<0.001), but no change in daily poly-unsaturated fatty acid (PUFA) intake over time. Compared to baseline, there was a significant increase in serum LDL at 1 month (p<0.001) and 2 months (p<0.01) and an increase in serum TC at 1 month (p<0.01) but not 2 months. DISCUSSION/SIGNIFICANCE OF IMPACT : Despite a significant increase in total fat, saturated fat and mono-unsaturated fat intake as well as an increase in total cholesterol and LDL levels following MAD initiation, dietary fat composition appears to minimally predict serum lipid values in the short term. CONFLICT OF INTEREST DESCRIPTION: Tanya McDonald has received speaking honoraria from Nutricia North America. Bobbie Henry-Barron receives grants from Johns Hopkins Institute for Clinical and Translational Research (ICTR) which is funded in part by Grant Number UL1 TR 001079 from the National Center for Advancing Translational Sciences (NCATS) a component of the National Institutes of Health (NIH), and NIH Roadmap for Medical Research, Nutricia and Vitaflo. Diane Vizthum receives grants from the Johns Hopkins Institute for Clinical and Translational Research (ICTR) which is funded in part by Grant Number UL1 TR 001079 from the National Center for Advancing Translational Sciences (NCATS) a component of the National Institutes of Health (NIH), and NIH Roadmap for Medical Research. Mackenzie C. Cervenka has received grant support from Nutricia North America, Vitaflo, Army Research Laboratory, The William and Ella Owens Medical Research Foundation and BrightFocus Foundation. She receives speaking honoraria from LivaNova, Epigenix, Nutricia North America and the Glut1 Deficiency Foundation and performs consulting with Nutricia North America and Sage Therapeutics and receives Royalties from Demos Health.

